# Cost of persistent asthma in Italy

**DOI:** 10.1186/s40248-016-0080-1

**Published:** 2016-12-16

**Authors:** Roberto W. Dal Negro, Chiara Distante, Luca Bonadiman, Paola Turco, Sergio Iannazzo

**Affiliations:** 1 National Centre for Respiratory Pharmacoeconomics and Pharmacoepidemiology, Verona, Italy; 2 SIHS Health Economics Consulting, Torino, Italy; 3 Research & Clinical Governance, Verona, Italy

**Keywords:** Asthma Cost, Asthma impact, Bronchial Asthma, Pharmacoeconomics

## Abstract

**Background:**

Asthma is a common disease of the airways with a significant burden for the society and for patients’ quality of life. The Social Impact of Respiratory Integrated Outcomes (SIRIO) study estimated a mean cost of 1,177.40 € per patient/year in Italy, in 2007. The aim of the present study was to update the cost of persistent asthma patients in Italy.

**Methods:**

An observational, retrospective, bottom-up analysis was carried out starting from the data base operating in the Lung Unit of the Specialist Medical Centre (CEMS), Verona (Italy), over the period June 2013-December 2015. Patients’ data were recorded over the 12 ± 2 months before the enrollment and during 12 ± 2 months of follow-up. The prospective was the Italian National Health Service and the broad Italian society. Clinical data were measured in terms of forced expiratory volume in 1 s (FEV_1_%) and number of relapses. Healthcare resources (namely; number of hospitalizations and/or ER admissions; number of visits; drug use and duration, and indirect costs) were recorded.

**Results:**

The cohort consisted of 817 patients with persistent asthma of different severity. They had a 42.96% male prevalence; a mean (±SE) age of 49.06 (±0.64) years; a mean 87.47% (±0.81) FEV_1_% pred. in baseline, and 69.16% of subjects had comorbidities. The mean (±SE) number of relapses was 0.91 (±0.09) per patient/year before the enrolment. After 12 months, FEV_1_% significantly improved by +6.31% (±0.45) from the corresponding baseline value (*p*  < 0.001). The number of relapses decreased of −0.46 (±0.09) (*p*  < 0.001). The estimated total annual cost per asthmatic patient was 1,183.14 € (±65.79 €) during the 12 months before the enrolment, and 1,290.89 € (±68.74 €) throughout the follow-up. The increase was mostly due to the significantly increased duration of therapeutic strategies. The costs of hospitalization, general practitioner and rescue medications were significantly decreased.

**Conclusions:**

The periodic update of cost analysis is a key to monitor the trend of main asthma outcomes and related expenditure over time. It allows to plan the most convenient actions in terms of prevention strategies and effective interventions, with the aim of optimizing the healthcare resources consumption and maximizing the impact on clinical outcomes and patients’ quality of life. The role of an appropriate pharmacological strategy still proves crucial in minimizing asthma morbidity and the corresponding socio-economic impact.

## Background

Asthma is a chronic disease of the airways which has a high impact on patients, their families, and society [1]. It is usually characterized by airway inflammation, occurrence of reversible airway obstruction of variable extent, and respiratory symptoms (namely wheeze, shortness of breath, chest tightness, and/or cough). Asthma severity is assessed based on the frequency of symptoms, the value of forced expiratory volume in 1 s (FEV_1_), and the variability of peak expiratory flow (PEF). On this basis, the severity of the disease is usually classified into four levels: mild intermittent, mild persistent, moderate persistent and severe persistent [2]. Airflow obstruction and symptoms change substantially after medication even if they can also change spontaneously over time. Patients can experience episodic flare-ups of asthma that may be life-threatening and cause a significant burden to patients and the society [2]. The disease also imposes a high socio-economic impact through loss of productivity of patients [1].

The World Health Organization (WHO) estimated 235 million people suffering from asthma worldwide [1]. In 2012, the National Institute of Statistics (ISTAT) reported that chronic disease of lower respiratory tract is the seventh cause of death in Italy [3]. Italian hospitalization data reported in 2013 618,052 discharges for principal diagnosis of respiratory system diseases (8% of the total discharges) (349,185 in males and 268,867 in females) [4].

The total cost of asthma is significant, and it reached the amount of 33.9 billion euro in Europe in 2011, with 19.5 billion € for direct costs, and 14.4 billion € for indirect costs [5]. In Italy, the Social Impact of Respiratory Integrated Outcomes (SIRIO) study investigated a sample of 485 patients with bronchial asthma, and estimated the total cost of 1,177.40 € per patient/year in 2007 [6].

The present study was aimed at estimating the economic burden of persistent asthma in order to define the current picture of health care resource consumption and update the cost of asthma patients from the Italian National Health Service (NHS) and the societal perspectives.

## Methods

The study was an observational, retrospective, bottom-up investigation of the clinical data and of healthcare resources carried out on asthma patients of different severity, referring to the Lung Unit of the Specialist Medical Centre (CEMS), Verona (Italy) over the period June 2013-December 2015. The duration of the follow-up analysis was 12 (±2) months.

The aim was to estimate the annual costs of persistent asthma patients in the perspective of the Italian NHS and the broad Italian Society. Data were obtained automatically and anonymously from the institutional database of the Lung Unit operating in the Specialist Medical Centre (CEMS), Verona, Italy. The data base was UNI EN ISO 9001–2008 validated, and the classic Boolean algebraic formula was used for selections [7]. Selection criteria were: patients suffering from persistent atopic or non-atopic asthma of both genders; age > 18 years; the availability of a data file covering both the observational periods, including all patient’s historical, clinical, pharmacological, and lung function data.

At baseline, demographic and clinical characteristics (such as: gender; age; smoking habit; predicted values of forced expiratory volume in 1 s (FEV_1_%); the presence of possible concomitants diseases, and the therapeutic strategy) were recorded throughout the previous 12 months. Atopic condition; perennial or seasonal rhinitis; conjunctivitis; rhino-conjunctivitis; sinusitis (with or without nasal polyps); dermatitis, and eczema were considered as comorbidities likely related to atopic asthma. Aspirin-intolerance was also recorded.

According to the study protocol, all selected patients had to be stratified by their asthma severity as assessed at the enrollment in terms of their FEV_1_% predicted values, such as: Group A, patients with FEV_1_ ≥ 80% pred.; Group B, FEV_1_ < 80 and ≥ 60% pred., and Group C, FEV_1_ < 60% pred.

Relapses (causing hospitalizations or not), occurred in the 12 (±2) months before the enrollment were recorded. FEV_1_% pred. was recorded together with the number of relapses also during the 12-month follow-up.

Health care resources consumed during the 12 (±2) months prior the enrollment, and throughout the following 12 (±2) months were calculated. Hospitalizations and Emergency Room admissions due to asthma relapses, together with the number of GP’s and lung physician’s visits, and the overall pharmacological consumption (drugs for treating both asthma and comorbidities) contributed to define the asthma direct costs.

The hospitalization cost was calculated as the mean cost for asthma relapse according to the National Diagnosis-Related Group (DRG) tariffs [8]. At present, the hospitalization cost due to asthma relapse is valued at 2,537 € (DRG 96) in the presence of relevant comorbidities, and at 1,832 € (DRG 97) in the absence of relevant comorbidities, respectively.

The GP’s visit cost was estimated in 15.17 € based on a published cost study inflated to Euro 2016 according to the ISTAT consumer price index [9, 10]. The cost of the specialist’s visit was 20.66 €, derived from the National Inpatient Tariffs (Code 89.7) [11].

The overall pharmaceutical cost was obtained by adding the cost of all respiratory drugs directly related to asthma treatment (i.e., the principal treatment, by daily dose and duration of administration); the cost of steroid and/or antibiotic courses, and of other rescue medications, and the cost of other drugs used for treating concomitant diseases, all carefully recorded over the two observational periods. All these pharmaceutical costs were estimated considering the ex-manufactory pack-prices [12].

Indirect costs were calculated as costs due to the number of work-off days or days of inactivity, considering the national hourly labour cost of 28 € [13], weighted by the number of days reported in each patient’s file.

All data from the overall sample and from the three subgroups of subjects were analyzed over time.

### Statistics

Mean value and standard error were calculated. *p* values refer to Wilcoxon rank-sum test. Statistical significance was accepted for *p* < 0.05.

## Results

The overall selected sample consists of 817 patients suffering from persistent asthma of different severity. Northern regions contributed for 45.8% (*n* = 374), while central regions for 14.5% (*n* = 119), and southern regions for 39.7% (*n* = 324) to their geographic distribution. The demographic and clinical characteristics of the overall cohort and of the three subgroups of patients recorded at baseline are summarized in Table 1. The overall male prevalence was 42.96%, while mean (±SE) age was 49.06 (±0.64) years, and mean (±SE) FEV_1_% pred. was 87.47 (±0.81). Active smokers were 8.2%, while ex-smokers 14.1%. The overall prevalence of comorbidities was relevant (69.16%), and atopic-related disorders were 56%. The overall mean (±SE) annual number of relapses during the 12 (±2) months before the enrollment was 0.91 per patient/year (±0.09).

**Table 1:** Baseline characteristics of the overall cohort and of the three subgroups of patients, according to their FEV_1_% predicted value

	Overall cohort	Group A FEV_1_ ≥ 80%	Group B FEV_1_ < 80 ≥ 60%	Group C FEV_1_ < 60%
*n*	817	508	202	107
male sex, %	42.96	43.90	41.09	42.06
age, years	49.06 (±0.64)	43.42 (±0.78)	57.46 (±1.16)	60.00 (±1.17)
FEV_1_%	87.47 (±0.81)	102.36 (±0.57)	70.71 (±0.41)	48.39 (±0.87)
Relapses, n,^a^	0.91 (±0.09)	0.75 (±0.09)	0.92 (±0.13)	1.15 (±0.21)
Presence of comorbidities, %	69.16	59.84	81.68	89.72

Mean (±SE, standard error)

FEV_1_%: forced expiratory volume in 1 s, predicted values

^a^Relapses with and without hospitalization, in the 12 ± 2 months before the study

After the 12 (±2) month-follow-up, the mean (±SE) FEV_1_% pred. was 93.79% (±0.70) in the overall sample, by a significant difference of +6.31% (±0.45) from the corresponding baseline value (*p*  < 0.001). In particular, the mean (±SE) FEV_1_% was 103.92 (±0.60) in Group A; 83.92% (±1.00) in Group B, and 64.34 (±1.73) in Group C, respectively (Table 2).

**Table 2:** Clinical data after 12 ± 2 months of optimized therapy

	Overall cohort	Group A FEV_1_ ≥ 80%	Group B FEV_1_ < 80 ≥ 60%	Group C FEV_1_ < 60%
*n*	817	508	202	107
FEV_1_%	93.79 (±0.70)	103.92 (±0.60)	83.92 (±1.00)	64.34 (±1.73)
Relapses, n,^a^	0.45 (±0.03)	0.31 (±0.03)	0.62 (±0.08)	0.79 (±0.12)

Mean (±SE, standard error)

FEV_1_%: forced expiratory volume in 1 s, predicted values

^a^Relapses with and without hospitalization

The mean (±SE) annual number of relapses was 0.45 (±0.03)/patient in the overall sample; in particular, it was 0.31 (±0.03)/patient in Group A; 0.62 (±0.08)/patient in Group B, and 0.79 (±0.12)/patient in Group C, respectively. The mean difference in the relapse rate between the 12-month observational period and the 12-month before the enrollment was −0.46 (±0.09) (*p*  < 0.001) in the overall cohort.

The mean (±SE) number of days of inactivity was 2.24 (±0.29)/patient during the 12 months before the enrollment, and 2.11 (±0.25)/patient after 12 months of optimized therapy, by a difference of −0.13 (±0.34) (*p* = 0.30). During the 12 months prior to the enrollment, the mean (±SE) number of days of inactivity was 1.62 (±0.26)/patient in Group A; 2.94 (±0.67)/patient in Group B, and 3.83 (±1.32)/patient in Group C, respectively. After a 12- month observational period, the mean (±SE) number of days of inactivity was 0.86 (±0.14)/patient in Group A; 3.84 (±0.77)/patient in Group B, and 4.77 (±0.96)/patient in Group C, respectively. In particular, the number of days of absenteeism was lowered by 47% only in mild patients. The cohort of severe patients proved a much more scattered behaviour without any significant trend (*p* = ns).

### Health care resources

In the overall sample, the mean (±SE) annual number of hospitalizations/ER visits was 0.09 (±0.01)/patient over the 12 months before the enrollment, and 0.04 (±0.01)/patient after a 12-month observational period. The mean (±SE) annual numbers of GP visits/patient were 0.86 (±0.06) and 0.33 (±0.03) in the same periods, respectively, while those of the lung physician’s visits were 0.88 (±0.04)/patient and 0.98 (±0.04)/patient, respectively.

Furthermore, the mean (±SE) numbers. of courses of systemic steroids and/or of antibiotics, and/or of other rescue medications were 0.65 (±0.05) and 0.46 (±0.04)/patient during the 12 month-period before the enrollment and throughout the following 12 months, respectively (Table 3).

**Table 3:** Annual healthcare resource use/patient at baseline (during the 12 ± 2 months before the enrollment), and throughout the following 12 months in the overall sample

	Baseline	After 12 months	Difference	*p*
Hospitalizations	0.09 (±0.01)	0.04 (±0.01)	−0.05 (±0.01)	<0.001
GP visits	0.86 (±0.06)	0.33 (±0.03)	−0.53 (±0.06)	< 0.001
Specialist visits	0.88 (±0.04)	0.98 (±0.04)	0.10 (±0.06)	0.001
Rescue medication (courses of steroids, antibiotics, and other rescue drugs)	0.65 (±0.05)	0.46 (±0.04)	−0.19 (±0.06)	< 0.001

Mean (±SE, standard error)

*p* values refer to Wilcoxon rank-sum test

*GP:* general practitioner

**Fig. 1:**
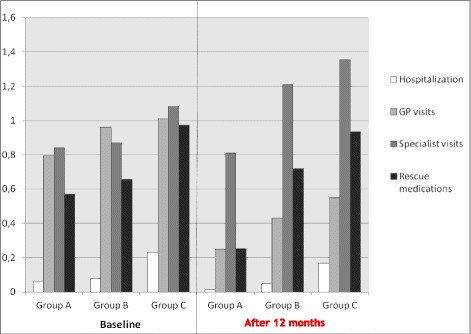
Annual healthcare resource use/patient at the baseline (during the12 ± 2 months before the enrollment study), and throughout the following 12 months in the three subgroups of patients of different asthma severity

The annual consumption of health care resources in the three subgroups of patients is reported in Fig. 1. The mean (±SE) annual number of hospitalizations/patient decreased by a mean difference of 0.05 (±0.01) (*p* < 0.001) in Group A; 0.03 (±0.02) (*p* = 0.224) in Group B, and 0.07 (±0.06) (*p* = 0.567) in Group C, respectively, during the following 12 months. The mean (±SE) annual number of GP visits/patient also decreased in the three subgroups of patients, by a difference of 0.55 (±0.07) (*p* < 0.001) in Group A; 0.53 (±0.13) (*p* < 0.001) in Group B, and 0.46 (±0.23) (*p* = 0.216) in Group C, respectively, in the same periods, while the mean (±SE) annual number of lung physician’s visits dropped by 0.03 (±0.06) (*p* = 0.516) in Group A; this number increased significantly in Group B and C by 0.34 (±0.11) (*p* = 0.001) and 0.27 (±0.23) (*p* = 0.004), respectively.

Finally, the mean (±SE) annual number of systemic steroid, and/or antibiotic courses, and/or other rescue medications decreased significantly during the following 12 months by 0.32 (±0.06) (*p* < 0.001) in Group A, while it did not change significantly in Group B (−0.04 (±0.23); *p* value = 0.548) and C (+0.06 (±0.13); *p* = 0.724), respectively.

### Costs

In the overall sample, the mean (±SE) annual total cost per patient was estimated of 1,183.14 € (±65.79 €) during the 12 months before the enrollment, and of 1,290.89 € (±68.74 €) at the end of the 12-month observational period. The mean difference between the two periods was significant, such as, +107.76 € (±71.62) (*p* < 0.001) (Table 4).

**Table 4:** Costs over the 12 months before the enrolment and at the end of the following 12 months in the overall sample (€/patient/year)

	Baseline	After 12 months	Difference	*p*
Hospitalization	218.06 (±29.02)	104.37 (±18.44)	−113.69 (±28.69)	< 0.001
GP visits	13.09 (±0.88)	5.06 (±0.42)	−8.03 (±0.91)	< 0.001
Specialist visit	18.18 (±0.89)	20.23 (±0.77)	2.05 (±1.16)	< 0.001
Rescue medication	19.19 (±1.41)	13.39 (±1.19)	−5.80 (±1.70)	< 0.001
Concomitant therapies	50.65 (±4.34)	60.20 (±4.90)	9.55 (±2.84)	ns
Respiratory therapies	636.68 (±19.87)	851.30 (±23.88)	214.62 (±21.55)	< 0.001
DIRECT COSTS	955.85 (±41.37)	1,054.56 (±37.20)	98.71 (±41.91)	< 0.001
Days of inactivity	227.29 (±42.59)	236.34 (±44.58)	9.05 (±50.88)	ns
INDIRECT COSTS	227.29 (±42.59)	236.34 (±44.58)	9.05 (±50.88)	ns
TOTAL COSTS	1,183.14 (±65.79)	1,290.89 (±68.74)	107.76 (±71.62)	< 0.001

Mean (±SE, standard error); GP: general practitioner

*p* values refer to Wilcoxon rank-sum test; ns: not significant

The mean (±SE) annual direct cost/patient was 955.85 € (±41.37 €) at baseline and 1,054.56€ (±37.20€) at the end of the following 12 months, respectively, by a mean difference of +98.71 € (±41.91€) (*p* < 0.001).

In particular, the mean (±SE) annual cost due to hospitalization/patient was 218.06 € (±29.02 €) at baseline and 104.37 € (±18.44 €) throughout the following 12 months, by a mean significant difference of –113.69 € (±28.69 €) (*p* < 0.001). Moreover, the mean (±SE) annual costs due to GP visits were 13.09 € (±0.88 €) and 5.06 € (±0.42 €)/patient in the same period, by a mean difference of –8.03 € (±0.91 €) during the following 12 months (*p* < 0.001). The corresponding mean (±SE) annual costs due to specialist visits were 18.18 € (±0.89 €) and 20.23 € (±0.77 €), respectively, with a mean difference of +2.05 € (±1.16 €) during the following 12 months.

Moreover, the mean (±SE) overall annual cost due to rescue medications was 19.19 € (±1.41 €) in the 12 months before the enrollment and 13.39 € (±1.19) per patient during the following 12 months, by a mean difference of –5.80 € (±1.70 €) (*p* < 0.001). The mean (±SE) annual cost due to daily respiratory drugs was 636.68 € (±19.87 €) over the first period and 851.30 € (±23.88 €) during the following 12 months, respectively, by a significant increase of +214.62 € (±21.55 €) (*p* < 0.001). To note that the mean treatment duration changed from 114.3 days (±1.11) throughout the 12 months before the enrollment to 287.4 days (±1.9) at the end of the following 12 months (*p* < 0.001), and that some expensive therapeutic options (namely, biological drugs) were implemented in some patients after the enrollment. The overall consumption of drugs for managing comorbidities slightly increased, even if not significantly, during the 12-month control period.

Finally, the mean (±SE) annual indirect costs were almost unchanged (such as 227.29 € (±42.59 €) over the period preceding the enrollment and 236.34 € (±44.58 €) during the following 12 months, by a mean difference of +9.05 € (±50.88 €) (*p* = 0.586).

Total costs calculated in the three subgroups of patients are described in Table 5. From a general point of view, all costs considered increased by the degree of asthma severity: as expected, the lowest costs were found in Group A and the highest ones in Group C, where some more recent and expensive therapeutic options were implemented in some patients. The difference between the total annual costs calculated after 12 months of optimized treatment and those calculated over the 12-month period before the enrollment in the three subgroups of patients highlights the significant drop in the hospitalization cost, as well as in those due to GP visits and to rescue medications during the following 12-month observational period.

**Table 5:** Total costs over the 12 months before the enrollment and at the end of the following 12 months in the three subgroups of patients of different asthma severity (€/patient/year)

	Baseline			After 12 months			Difference		
	Group A FEV_1_ ≥ 80%	Group B FEV_1_ < 80 ≥ 60%	Group C FEV_1 _< 60%	Group A FEV_1_ ≥ 80%	Group B FEV_1_ < 80 ≥ 60%	Group C FEV_1_ < 60%	Group A FEV_1_ ≥ 80%	Group B FEV_1_ < 80 ≥ 60%	Group C FEV_1_ < 60%
TOTAL COSTS	782.37 (±51.60)	1,592.08 (±159.67)	2,313.83 (±283.61)	709.30 (±30.19)	2,040.61 (±192.66)	2,636.76 (±286.23)	−73.07 (±51.19) (*p* < 0.001)	448.52 (±196.20) (*p* < 0.001)	322.93 (±316.64) (*p* 0.012)

FEV_1_%: forced expiratory volume in 1 s, predicted values

**Fig. 2:**
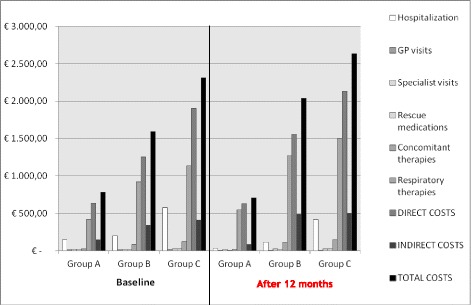
Costs during the 12 months before the enrollment and at the end of the following 12 months in the three subgroups of subjects of different asthma severity (€/patient/year)

In particular, the total costs were decreased by –73.07 € (±51.19 €) in Group A; and increased by +448.52 € (±196.20 €) in Group B, and by +322.93 € (±316.64 €) in Group C, all significantly (*p* < 0.05), at the end of the following 12 months (Fig. 2).

More in detail, the mean (±SE) annual cost due to hospitalizations changed from 150.10 € (±26.41 €) to 33.57 € (±12.68 €) in Group A; from 197.46€ (±47.61 €) to 115.12 € (±35.94 €) in Group B; and from 579.58 € (±155.22 €) to 420.20 € (±102.70 €) in Group C, respectively, (all *p* < 0.001).

The costs due to respiratory treatments significantly increased over the follow-up in the three subgroups of patients, and they changed by +127.11 € (±15.70 €) (*p* < 0.001) in Group A; by +353.38 € (±59.12 €) (*p* < 0.001) in Group B, and by +368.15 € (±90.92 €) (*p* = 0.002) in Group C, respectively.

The costs due to concomitant therapies significantly decreased over the 12-month control period in Group A, and they changed by −2.63 € (±1.77 €) (*p* < 0.032). These costs increased over the control period in the other subgroups of patients, and they changed by +30.39 € (±8.52 €) (*p* < 0.002) in Group B, and by +28.05 € (±11.20 €) (*p* = 0.299) in Group C, respectively.

## Discussion

Asthma imposes a high burden on patients and society due to its chronicity, loss of productivity, and increase in use of healthcare resources. We carried out the present observational retrospective analysis on persistent asthma patients with the aim of calculating and updating their annual costs from the Italian NHS and the broad Italian society.

In general terms, due to the extent, the general characteristics, and the wide geographical distribution of the sample, the total annual cost/patient calculated in the previous 12 months (such as before any intervention) can be regarded as an estimate approaching very closely to the national economic impact of persistent asthma, because actually reflecting the general approach within the territories.

From a general point of view, the overall severity of the disease, measured in terms of changes in FEV_1_% predicted and in number of relapses at the end of the 12 ± 2 month-follow-up, was improved when compared to that measured at baseline, which was mirroring the conditions assessed over the 12 ± 2 months before the enrollment. In the same way, likely due to a more careful and appropriate treatment provided by the specialist Center, the mean annual number of hospitalizations was significantly decreased, as well as that of GP visits, and the overall use of rescue medications. On the other side, the annual total direct cost/patient rose from 955.85 € before the enrollment to 1,054.56 € over the follow-up, by a significant difference of +98.71 €. This was particularly due to the increase in the cost for respiratory treatments (+214.62 €), which also were of a substantially longer duration than in the previous 12 months. This cost was partially compensated by the substantial decrease in the hospitalization cost (−113.69 €), and by the optimization of morbidity of persistent asthma during the same period.

In 2007, the SIRIO study estimated a mean total annual cost/patient of 1,177.40 € in Italy [6]. In that study, a cohort of 485 asthmatic patients of different severity (intermittent asthma 26.2%; mild persistent 37.1%, moderate persistent 29.5%, and severe persistent asthma 6.6%) was analyzed. When compared to the SIRIO cohort, the sample of patients investigated in the present study was much bigger (817 vs 485 patients);it had the same overall mean age (i.e., 49 years), the same female prevalence (i.e., close to 60%), and the same duration of the investigational period (such as, 12 month before the enrollment and 12 months of control).

In the SIRIO study, the calculated total annual cost/patient was of 1,434.02 € before the follow-up, which dropped to 1,177.40 € after the follow-up. To point out that, the present mean total annual cost/patient resulted 1,183.14 € at the enrollment, and it changed by −17.5% when compared to the one calculated in 2007. In other words, this change represents a true, substantial drop in the mean asthma cost/patient because, unlike SIRIO study, intermittent asthma (such as the less expensive clinical manifestation of asthma) was not considered in the present study.

It should be also taken into account that, even if other costs were also considered in the SIRIO study (namely, costs for diagnostic tests; costs due to possible side effects; to environmental preventive treatments, and to alternative treatment, by an overall contribution of 13.2% to total cost), in the present study the cost of expensive biological asthma treatments (unavailable in 2007) were included, and their relative contribution to total asthma cost largely compensates that of the other minor costs previously reported.

Moreover, the trend of costs due to respiratory treatment and those to hospital admissions in the SIRIO study were similar to the corresponding costs calculated in the present study.

Actually, the cost/patient due to principal respiratory treatments (about 61% of the total costs) increased at the end of the follow-up. In absolute terms, the cost/patient for respiratory therapies changed from 398.79 € at baseline to 717.06 € at the end of the 12-month observational period (such as, +318.27 €) in 2007, while this difference was of + 214.62 € in the present study.

The cost due to hospital admissions dropped by 52.1% in the present study after the 12-month period of control, while it dropped by 65% in the SIRIO study: this trend confirms and highlights the crucial role of the treatment optimization by lung physicians over the period.

The cost/patient for concomitant therapies at the end of follow-up increased of +9.55 € in our study (about 5% of the total cost), and similarly of +10.30 € in the SIRIO study (about 6% of the total cost).

Indirect costs calculated at baseline in the present study were comparable to those of the SIRIO study, and remained almost unchanged over the control period, even if a substantial drop of work days off was observed in mild asthmatics. On the other hand, the unchanged impact on absenteeism observed in severe asthma patients can be likely due to the introduction of biological asthma treatments for these subjects. Actually, even if much convenient in terms of hospitalization rate and disease control, these therapeutic options require to be periodically administered exclusively in an hospital setting, thus these procedures usually require a corresponding work off period.

As in the SIRIO study, also present data confirm the parallel, increasing trend of health care consumption and costs according to asthma severity. Particularly in Group A and B, the hospitalization rate, the frequency of GP and specialist visits, and the number of rescue medication courses proved lower when compared to those of Group C. Obviously, the corresponding costs calculated in these subgroups had a similar trend.

All these results are in line with previous studies that had demonstrated the parallel trend of increasing severity of asthma and of increasing costs for patient and society [6, 14]. Actually, a European study carried out over 1999–2002 in 11 European countries, including Italy, estimated an average annual cost/patient of 1,583 €, such as, of 509 € per patient with controlled asthma, and of 2,281 € per patient with uncontrolled asthma, respectively [14].

The most relevant results emerging from the present up-date of annual asthma cost proved that total annual cost/patient is significantly decreased when compared to the one calculated in 2007 (−17.5%). This economic outcome is likely due to the progressively increased awareness of asthma burden, and to the consequent more appropriate therapeutic approach in recent years.

The progressive, substantial decrease in hospital admissions and incidence of asthma relapses tends to strongly support this hypothesis. In other words, the morbidity of asthma looks much more contained than in the past. In particular, the hospitalization rate of asthma progressively dropped from 22 to 5% during these 8 years, with a consequent dramatic drop in asthma costs. The widespread adoption of GINA guidelines, continuously updated, likely played a crucial role from this point of view. These data were confirmed a few years ago even at European level, when it was further proved that a substantial cost savings could be obtained through a proper management of asthma [15].

From this point of view, also present data tend to further emphasize the crucial role of the appropriateness of the therapeutic strategy in persistent asthma patients. This point is highlighted by the substantial increase in the pharmaceutical costs, in particular those due to respiratory drugs. Even if already significantly higher at baseline, this cost reached 66% of total asthma cost at the end of the follow-up in the present study, such as a cost higher by 6.2% than the one calculated in 2007. The longer duration of daily treatments, together with the use of biological treatments previously unavailable, greatly contributed to this increase of pharmaceutical asthma cost.

In agreement with a survey which compared the trend of pharmaceutical and hospitalization costs calculated in nine studies dealing with asthma pharmacoeconomics in Italy, the increase in resources spent for appropriate pharmacological interventions once again strictly corresponds to the substantial decline of costs related to hospital admissions [15].

The main limit of the present study is related to its mono-centric design. Even if geographically well distributed, all patients were managed at the Lung Unit of the Specialist Medical Centre (CEMS), Verona (Italy), during the following 12 months. As a consequence, despite the large cohort (817 patients), the outcomes achieved following asthma treatment cannot be considered as fully mirroring the effects of the general approach to asthma management. Another limit of the study can be ascribed to the use of national tariffs as a proxy of unit costs of the healthcare resources. This was related to the clinical nature of the database, which was not reporting actual healthcare costs.

## Conclusions

Persistent asthma has a significant impact on patients’ quality of life, and economic burden for the society. The periodic update of cost analysis is important in order to monitor the trend of main outcomes, and to allow the most appropriate interventions for preventing and treating the disease.

Besides that of prevention of risk factors, the role of an appropriate pharmacological strategy is crucial to minimize hospital admissions, ER visits, asthma morbidity, and the corresponding socio-economic impact.
